# Supplementary figures and images for: And growth on form? How tissue expansion generates novel shapes, colours and enhance biological functions of Turing colour patterns of Eukaryotes
Source: PLoS One. 2025 Feb 3;20(2):e0305921. doi: 10.1371/journal.pone.0305921 (PMC11790173; doi:10.1371/journal.pone.0305921)

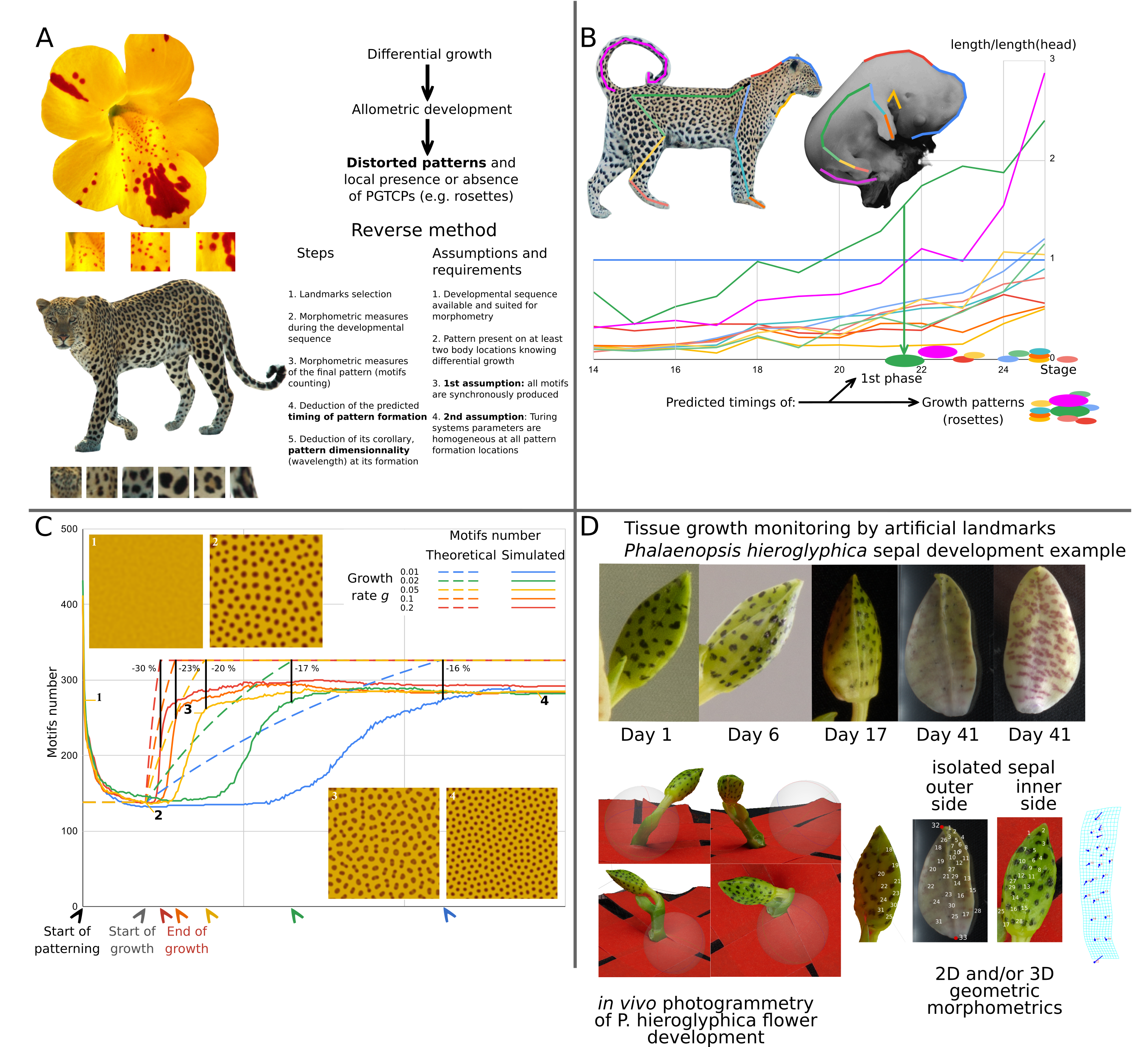

Supplement: S1 Fig — (A) Left: examples of distortions in classic Turing pattern (Mimulus sp.) and a PGTCP (Panthera pardus) Right: reverse method principles and assumptions. (B) Dynamics of relative size of various distances to head size during development of the cat (Felis silvestris stages 14 to 23) and leopard (Panthera pardus, stage 24 and 25) and predicted classic and PGTCPs pattern formation. (C) Simulation of dynamics of motifs number during pattern formation under tissue growth. Y-axis: number of motifs simulated at different growth rates (solid lines), compared to the theoretical number of motifs (dashed lines). Up left and down right: 4 details of pattern phenotype through growth for g = 0.05, at different times (1: early, 2: pattern at the start of growth, 3: pattern just before growth stops, 4: late pattern at the equilibrium). (D) Tissue growth monitoring with hand-written landmarks on the tissue, example of Phalaenopsis hieroglyphica sepal outer epidermis. Upper panel: Sequence of landmarked flower bud. Lower panel: Left, four views extracted from the photogrammetry model of P. hieroglyphica flower bud (at Day 6). Right, preliminary 2D geometric morphometrics landmarks assessment and comparison between young stage (Day 6, 1st and 3rd pictures) and final stage (Day 41, 2nd picture). Fourth picture: grid deformation between young and late stage after Procrustes analysis. Pictures sources: Patrick Giraud 2006 https://commons.wikimedia.org/wiki/File:Namibie_Etosha_Leopard_01edit.jpg, Hugo.arg 2007 https://commons.wikimedia.org/wiki/File:Mimulus001.JPG. (TIF) [file pone.0305921.s001.tif]

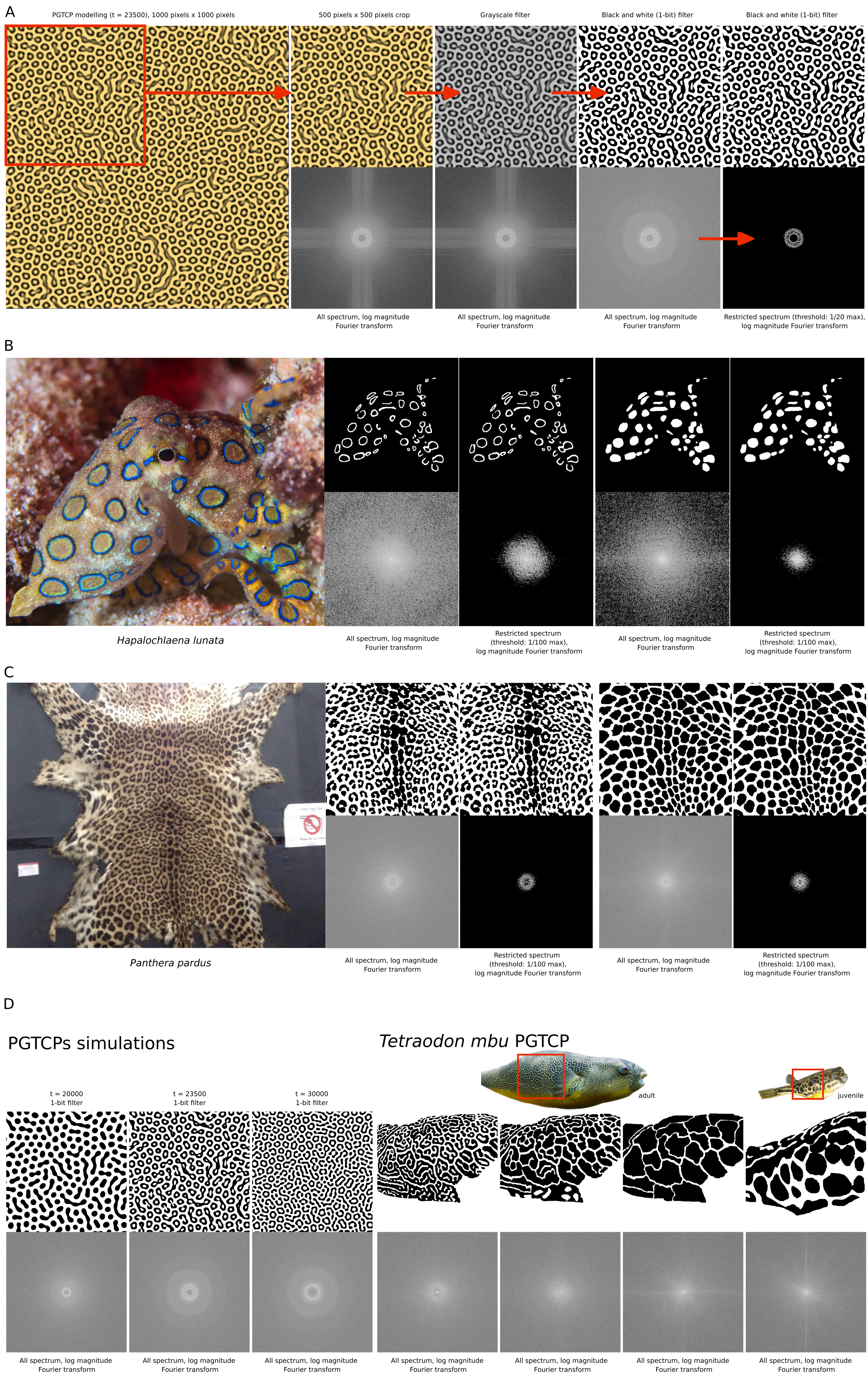

Supplement: S2 Fig — (A) Filters applied to PGTCPs simulations output to obtain a 1-bit image and the respective frequency magnitude spectra. (B) DFT of blue-ringed octopus Hapalochlaena lunata PGTCPs (first two panels) and their modified version without PGTCP (last two panels). (C) DFT of leopard (Panthera pardus) flastskin PGTCPs (first two panels) and their modified versions without PGTCP (last two panels). (D) DFT of simulated PGTCPs (first three panels, log magnitude, no threshold) and Tetraodon mbu PGTCPs (last four panels) and their modified version without PGTCP (last two panels, log magnitude, no threshold). Pictures sources: Rickard Zerpe, 2019 https://commons.wikimedia.org/wiki/File:Greater_blue-ringed_octopus_(Hapalochlaena_lunulata)_(48272090161).jpg, SagarPaudel68 2016 https://commons.wikimedia.org/wiki/File:Leopard_skin.jpg. (TIF) [file pone.0305921.s002.tif]

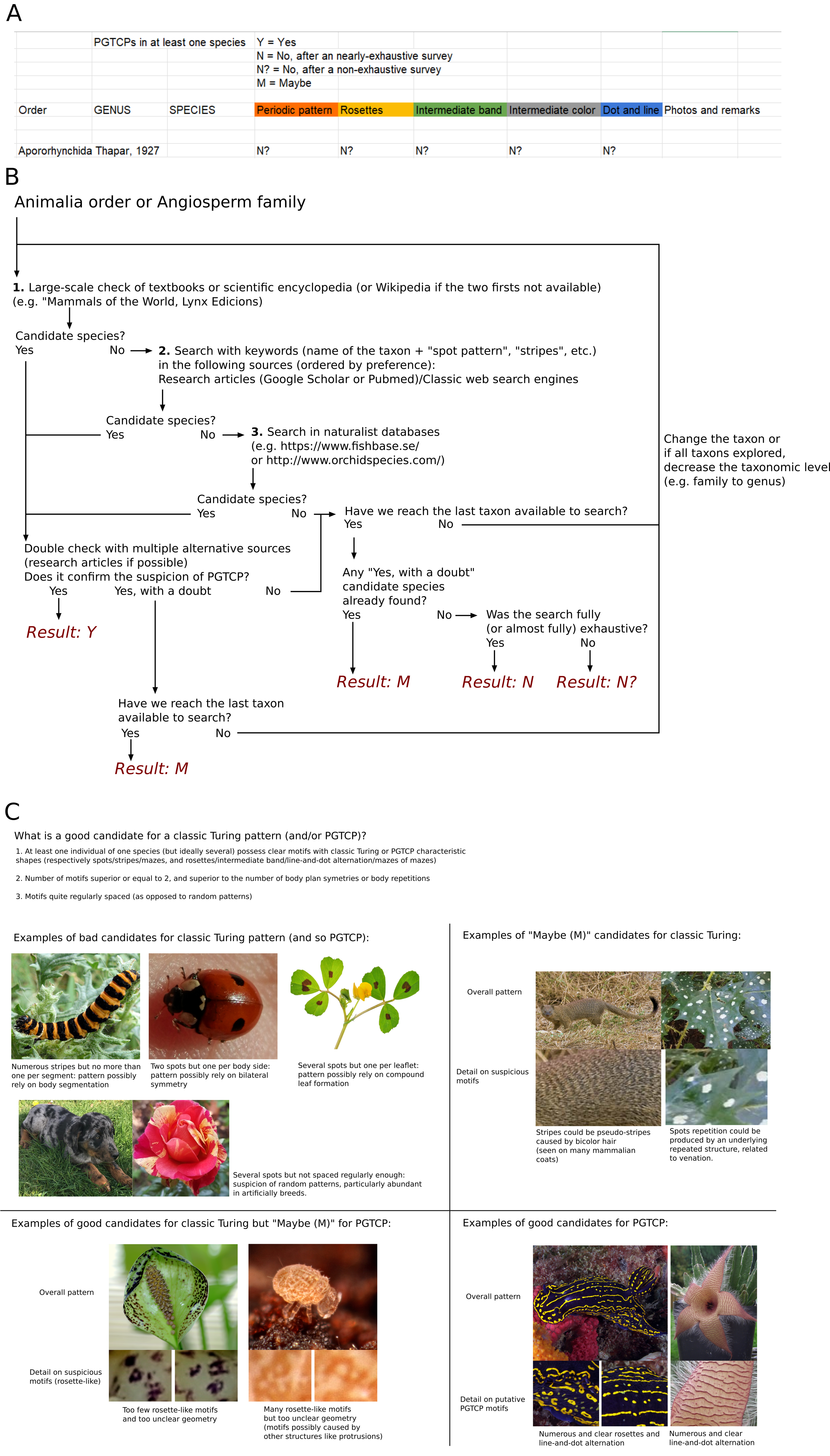

Supplement: S3 Fig — (A) Screenshot of PGTCP database. (B) Classic Turing and PGTCP survey flowchart. (C) Criteria and examples of candidates. A minimal number of three motifs is required to consider it as a good candidate for a PGTCP, as one or two motifs patterns might be produced by other mechanisms than the Turing system. Pictures sources: Derek Keats 2011 https://commons.wikimedia.org/wiki/File:Stapelia_gigantea_(5538239090).jpg, Greg McFal 2010, https://fr.wikipedia.org/wiki/Fichier:Regal_Sea_Goddess_Nudibranch.jpg. (TIF) [file pone.0305921.s003.tif]
