# Supplementary material for: And growth on form? How tissue expansion generates novel shapes, colours and enhance biological functions of Turing colour patterns of Eukaryotes
Source: PLoS One. 2025 Feb 3;20(2):e0305921. doi: 10.1371/journal.pone.0305921 (PMC11790173; doi:10.1371/journal.pone.0305921)
Supplement: Attachment — Submitted filename: Review_of_PONE_D_23_43374.pdf [file pone.0305921.s004.pdf]

Review of PONE-D-23-43374: And Growth on Form? How tissue expansion generates novel shapes, colours and enhance biological functions of Turing colour patterns of Eukaryotes.

Andrew L. Krause, Durham University, UK*

The author explores the role of domain growth in modulating Turing patterns, leading to 'non-classical' structures like stripes and dots, as well as rosettes. They survey a wide range of taxa from different phylogenies of Eukaryotes, finding that some lineages have variation in the occurrence of 'classical' Turing patterns (spots, stripes, mazes), as opposed to 'putative-growth Turing color patterns' (PGTCPs). Beyond these classifications, they build a simple model of a growing reaction-diffusion system, and perform a range of analyses between the observed morphologies and the simulated patterns. They include a nice discussion of plausible mechanisms regarding pattern fixation or disturbance, providing some interesting hypotheses regarding the role of growth (and other factors, such as memory and cell-fate fixation) in originating a diversity of patterns. They also consider a mechanism-based way of understanding the origin of these patterns given morphometric data.

Overall I find the topic interesting, and the core ideas of surveying wide taxa and matching observations with plausible theory very interesting. However, I have a number of serious concerns about the theoretical aspects of the work, largely to do with how the mathematical models are set up and how they are interpreted. I think the ideas in this paper are interesting, but from my point of view much more work is needed to make a significant contribution to the literature. Much of the analysis is broad but shallow, with some important caveats in reference to prior work on growth, pattern robustness, etc. Below I outline a number of suggestions for improvement. It should be noted that I do not think the authors must address *every* comment below in copious detail – rather, I feel they should consider telling a deeper story with more significant insights, focusing either less on specific mechanisms (and hence more qualitatively on the observations), or more narrowly justifying a specific mechanism such as growth in the formation of complex patterns.

Key Comments

1. **Growth is an Inadequate Mechanism for Pattern Modulation:** As far as I understand the current manuscript, the author ascribes patterns which do not fall into a classification of spots or labyrinthine structures (stripes or mazes) as putatively due to growth modulating a Turing (reaction-diffusion) mechanism on the level of gene expression (or plausibly cell phenotypes or another mesoscopic structure). However, growth itself requires a fairly precise mechanism of fixation to transform a reaction-diffusion pattern into a fixed morphological structure. This fixation is nontrivial. In fact, even just considering transient pattern formation alone (i.e. allowing a modeller to 'fix' a transient pattern at any stage), one can essentially generate any possible observed pattern given freedom over the nonlinearities and initial conditions.

   Perhaps more importantly, there are many other mechanisms which can also generate all of the 'non-classical' patterns described by the author. In particular, work combining ideas of positional information and Turing patterning (Green and Sharpe, 2015; Krause et al., 2020) emphasises that spatial modulation and simple 'pre-patterns' can generate structures such as rosettes and stripe-to-spot transitions within the domain. See Woolley et al. (2021) for a demonstration of how spatial and/or temporal forcing can give rise to essentially any pattern or sequence of observed patterns in reaction-diffusion systems. Even in spatially homogeneous (unforced) systems, one can find a huge range of different pattern behaviours, depending

*By signing this review, I waive my right to anonymity.

on the system's nonlinearities (presumptive reaction kinetics) and initial conditions; see (Pearson, 1993; Kondo and Miura, 2010) for several examples.

Overall, I am not at all convinced that structures such as rosettes must be due to growth, as they cannot be from growth alone but must involve a carefully-time fixation mechanism for them to survive beyond the embryonic stage. Additionally, I think there are several other plausible hypotheses regarding pattern modulation that more parsimoniously provide plausible mechanisms for such 'non-classical' patterns. I like the database that the author has built, and I do think such classification is valuable, but I would be substantially more hesitant in claiming that growth is the key modulator of patterning.

2. **Implementation of Domain Growth:** The model presented in (20) and implemented by the author in Processing neglects an important physical effect of growth. Namely, changing only the diffusion coefficient does not produce equations which satisfy conservation of mass as the domain size changes. To see this, note that changing the diffusion coefficient does not change uniform concentrations/spatially homogeneous equilibria. Assume such an equilibrium is in a domain which is growing. Then additional mass must be produced in order to maintain the same concentration everywhere on a larger region of space. This was an error in the original edition of the book (Murray, 2004), though was corrected by Crampin et al. (1999, 2002) and many others since. See, for example, the models in Seirin Lee et al. (2011); Van Gorder et al. (2021); Krause et al. (2023) which all include an extra term referred to as 'dilution.' For sufficiently slow growth, this term will be small, but it will still have an impact on the transition between patterns, the stability of patterns, etc.

   In the case of a domain given by $x \in [0, L(t)]$ which is growing uniformly in time, then the equations the author should instead simulate look like:

$$\frac{\partial \boldsymbol{u}}{\partial t} = \underbrace{\frac{1}{L^2}\boldsymbol{D}\nabla^2}_{\text{Diffusion}} \boldsymbol{u} + \underbrace{\boldsymbol{f}(\boldsymbol{u})}_{\text{Reactions}} - \underbrace{\frac{\dot{L}}{L}}_{\text{Dilution}}, \quad \dot{L} = \frac{dL}{dt},$$

   where $\boldsymbol{u}$ is a vector of concentrations, $\boldsymbol{D}$ is the matrix of diffusion coefficients, $\boldsymbol{f}$ are the reaction kinetics, and the final term arises due precisely to this domain growth effect. A very rudimentary version of the author's model with domain growth can be found at the URL in this footnote[1] using the Visual PDE website for simulating interactive PDE-based models. I don't think this exactly captures the physics, but one can see the growth-rate dependent behaviour due to this dilution term by increasing the growth rate parameter r – above some threshold, patterning is suppressed. Perhaps more importantly, the author can explore many of the simulations on this website that exhibit a variety of Turing-like patterns and related mechanisms of pattern formation.

3. **Connection to the Theoretical Literature:** As suggested above, I think the author has done an excellent job surveying a range of biological literature, but has missed crucial insights discussed in more theoretical papers. In particular, the original work of Crampin et al. (1999) was to ameliorate robustness issues in simple models of pattern formation; see Maini et al. (2012) for discussion of these issues, and the only partial progress made by introducing growth. As noted above, a major disadvantage of reaction-diffusion models on growing domains is how to 'fix' the pattern in time, providing another degree-of-freedom which needs tuned and invoking additional underlying mechanisms. Robustness is one of the key challenges even for simple/classical patterns; see Scholes et al. (2019) for further discussion of this point. I think one has to tread carefully in describing robust complex behaviour, as sufficiently complicated models can exhibit almost anything we'd like to see.

[1] https://visualpde.com/sim/?options=N4IgRgqiBcIHYFMCuBbAhnOIA04BqM8y6mO4AboQMxkDGYMALgE5IK62HkCWz3AJtwDOZfhCiwArAAZReArACMogOo[illegible]
aQAIAMgC-fQBPI1CLGztHFzdPHOOQfkpoFjYtRhhpADppfMUOCRAnADYbRVKjMARGNC9Y5wAlAEEAOQARNvtcxQ86BRLK6tr6xtbO7t7+
3ABrQgBlPQAHPwBePOUAbhq6tckthGWhNdzpcy3mNe27dc3LC7J0anMn7kJX3BRuOAANQiM+gMDzecAAmgCwiDcMtCABhJBCRgAexQZGYxSWy1iVSQ9nIHiMbiQNnizj6[illegible]
NJ7nJDXQBms5GwOhmIGY6lgSSEsJFOU+ICEAEcmKwEABfIA

Other Comments

4. The author states that Turing patterns have been observed *in vivo* and are responsible for many structures observed in biology. I would argue that almost all cases like this as presumptive; typically far more than two genes are implicated in these studies, and it is not perfectly clear that the mechanism is one of activator-inhibitor type. While the mechanism is plausible, I would be more cautious about its ubiquity across Eukaryotes. Even in work I was involved in (reference (13)), I think we are somewhat cautious to note that the reaction-diffusion mechanism is plausible, but that there are distinct modulations of the simple theory not entirely captured by simplistic two-species models. As an example, there has been recent work unraveling the more complex pattern formation in Zebrafish that goes beyond simplistic activator-inhibitor thinking (Bullara and De Decker, 2015). Even Shigeru Kondo, who authored reference (10), has since suggested important caveats to any simple reaction-diffusion model of Zebrafish (Kondo et al., 2021).

5. Line 66 is missing a paren around "(spots stripes and not much more)." I would also suggest that reaction-diffusion systems can exhibit an enormous diversity of patterns and structures when one moves away from the classical minimal model systems (Pearson, 1993; Woolley et al., 2021).

Minor Comments/Typos

6. Many of the references are incorrect and inconsistently typeset. For example, (29) is missing an author and is written in capital letters unlike nearby references.

7. The URL "eukolorpatterns.cnrs.fr" is misspelled with an @ symbol rather than a . before "fr."

NB: The manuscript already cites many references, so please do not feel the need to include any of the references I have added (especially any I am an author on). Rather, I am merely hoping the author considers some of the perspectives presented in these papers which may have been overlooked.

References

D. Bullara and Y. De Decker. Pigment cell movement is not required for generation of turing patterns in zebrafish skin. *Nature communications*, 6(1):6971, 2015.

E. Crampin, W. Hackborn, and P. Maini. Pattern formation in reaction-diffusion models with nonuniform domain growth. *Bulletin of mathematical biology*, 64(4):747–769, 2002.

E. J. Crampin, E. A. Gaffney, and P. K. Maini. Reaction and diffusion on growing domains: scenarios for robust pattern formation. *Bulletin of mathematical biology*, 61(6):1093–1120, 1999.

J. B. Green and J. Sharpe. Positional information and reaction-diffusion: two big ideas in developmental biology combine. *Development*, 142(7):1203–1211, 2015.

S. Kondo and T. Miura. Reaction-diffusion model as a framework for understanding biological pattern formation. *science*, 329(5999):1616–1620, 2010.

S. Kondo, M. Watanabe, and S. Miyazawa. Studies of turing pattern formation in zebrafish skin. *Philosophical Transactions of the Royal Society A*, 379(2213):20200274, 2021.

A. L. Krause, V. Klika, T. E. Woolley, and E. A. Gaffney. From one pattern into another: Analysis of Turing patterns in heterogeneous domains via WKBJ. *Journal of the Royal Society Interface*, 17:20190621, 2020.

A. L. Krause, E. A. Gaffney, and B. J. Walker. Concentration-dependent domain evolution in reaction–diffusion systems. *Bulletin of Mathematical Biology*, 85(2):14, 2023.

P. K. Maini, T. E. Woolley, R. E. Baker, E. A. Gaffney, and S. S. Lee. Turing's model for biological pattern formation and the robustness problem. *Interface focus*, 2(4):487–496, 2012.

J. D. Murray. *Mathematical Biology. II. Spatial models and biomedical applications.* Interdisciplinary applied mathematics. Springer, New York, 2004.

J. E. Pearson. Complex patterns in a simple system. *Science*, 261(5118):189–192, 1993.

N. S. Scholes, D. Schnoerr, M. Isalan, and M. P. Stumpf. A comprehensive network atlas reveals that turing patterns are common but not robust. *Cell systems*, 9(3):243–257, 2019.

S. Seirin Lee, E. A. Gaffney, and R. E. Baker. The dynamics of turing patterns for morphogen-regulated growing domains with cellular response delays. *Bulletin of mathematical biology*, 73:2527–2551, 2011.

R. A. Van Gorder, V. Klika, and A. L. Krause. Turing conditions for pattern forming systems on evolving manifolds. *Journal of mathematical biology*, 82:1–61, 2021.

T. E. Woolley, A. L. Krause, and E. A. Gaffney. Bespoke turing systems. *Bulletin of Mathematical Biology*, 83(5):1–32, 2021.
