# Supplementary material for: And growth on form? How tissue expansion generates novel shapes, colours and enhance biological functions of Turing colour patterns of Eukaryotes
Source: PLoS One. 2025 Feb 3;20(2):e0305921. doi: 10.1371/journal.pone.0305921 (PMC11790173; doi:10.1371/journal.pone.0305921)
Supplement: Attachment — Submitted filename: Response to Reviewers.docx [file pone.0305921.s005.docx]

To : the Editors of PLOS One	At Rennes, March, 28th 2024

Subject : Rebutal letter

Dear Editors,

I would like to thank the reviewers for their generous comments on the manuscript and I have edited the manuscript to address their concerns. I believe and hope that the manuscript is now suitable for publication in PLOS One.

Please find below my answers to every point mentioned by the reviewers. Their reviews are in bold and the answers in yellow-brown. Quotes of corrected texts are in yellow-brown and in italics.

I remain at your disposal for any information,

Very sincerely,

Dr. Pierre Galipot

ECOBIO

Université de Rennes - Campus Beaulieu

Rennes, FRANCE

***Reviewer #1***

***“My biggest point of this is that I’m still not really sure what the difference between a classical and a PGTCP is. The best definition I can see appears on page 11, which is too far into the paper. So I spend a long time not really understanding what the paper is talking about.***

***Saying that, my understanding is that a classical Turing system is a model of interacting agents where the interactions, movement mechanisms and domain features are all static, whereas a PGTCP is anything else.***

***From this point I’m not really sure as to the point of the paper.”***

Thanks for this useful comment.

I added a complete definition of PGTCPs in the introduction section.

-> lines 87 to 91 “*Here, we consider a particular color pattern as a PGTCP when it possess all of these characteristics: i) periodic, at least at the local scale, ii) non-based on a pre-existing periodicity (e.g. body segments or petals), iii) with at least three motifs (e.g. three stripes or rosettes) and iv) with at least one motif not belonging to the classical range of Turing geometries (dots, stripes, mazes), i.e. line-and-band alternations, rosettes for example.”*

***“There are papers out there which already highlight the diversity of Turing patterns***

***@Article{Krause-2020-FOP,***

***[...]***

***@Article{Krause-2018-HIS,[...]”***

Thank you for these references.

I will not comment on all parts of these articles, but only on what concerns PGTCPs. The first article (Krause 2020) proposes a way to produce rosette patterns of jaguar without necessarily growth (Figure 1), from a complex pre-pattern. I agree that some examples of PGTCPs might not be produced by growth in nature.

Anyway, the purpose of our article was not to claim that growth was the only way to produce PGTCPs, but the most likely for the majority of examples. We produced several types of arguments summarized in Figure 3.

As far as I understand the second article, it does not completely deal with PGTCPs formation.

To emphasize on the fact that growth is not the only way to produce PGTCPs, we add explanations in the introduction parts, and references, including (Krause 2020).

-> lines 75 to 77 “*Most of those models perfectly reproduced leopard rosettes by adding the most probable missing ingredient: tissue growth during pattern formation. Other types of missing ingredients have been described, like complex pre-patterns (21).”*

***“Moreover, the paper ignores a whole section of mechanisms through the application of noise to the systems, which also include growth.***

***@Article{Woolley-2011-PSS,***

***Title = {Power spectra methods for a stochastic description of diffusion on deterministically growing domains},***

***[...]”***

***“@Article{Woolley-2011-PSS2,***

***Title = {Influence of stochastic domain growth on pattern nucleation for diffusive systems with internal noise},***

***URL = {***[***http://link.aps.org/doi/10.1103/PhysRevE.84.041905***](http://link.aps.org/doi/10.1103/PhysRevE.84.041905)***}***

***[...]***

Thank you for bringing these articles to my attention.

These articles show another effect of growth in spatially heterogeneous patterns formation. Without Turing systems and only thanks to growth and stochasticity, the authors showed that heterogeneous patterns could arise. The resulting patterns are heterogeneous like Turing ones and PGTCPs but lack periodicity and some particular geometries, they are then not sufficient to explain the diversity of patterns evoked in our article.

***@Article{Woolley-2012-EIS,***

***Title = {Effects of intrinsic stochasticity on delayed reaction-diffusion patterning systems},”***

Thank you for bringing this article to my attention.

The authors showed that taking stochasticity into account could accelerate in some cases Turing pattern formation, compared to classic deterministic models. Growth is not simulated in this article so it is difficult to assess a particular role of stochasticity in PGTCPs formation.

***“Finally, there's even work on how to design models to ensure that you get patterns that you want***

***@Article{Woolley-2021-BTS,”***

This article proposes a nice and pedagogical method on how to build a pattern with particular desired motifs (spots, stripes or labyrinthine) from a desired set of ranges of Turing parameters. Nevertheless, it does not encompass PGTCPs motifs evoked in our paper (rosettes, intermediate colors, etc.) even if the juvenile of Tapir, presenting alternation of stripes and dots is shown in Figure 7. More precisely, simulation of coexistence of stripes and spots are shown (Figure 6), but not alternation of stripes and spots. In our paper, we propose that growth is not needed to explain simple coexistence but on the contrary is needed to produce an alternation. Therefore, this article does not sufficiently cover PGTCPs formation.

***“All in all, this is a nice biological review on the diversity of applications of extending Turing systems. This is needed for the biological community as well as for the mathematical community, but I don’t really see a conclusion beyond Turing systems can account for patterns beyond stripes, spots and labyrinthine if the mechanism is complicated in some manner… which we already knew. Thus, I suggest the author tries to find a stronger conclusion, or some different insight to finish on.”***

Thanks for this important remark. To better explain the purpose of the article and its conclusions, I rephrase some parts to be clearer:

-> lines 103 to 110: *“In summary, we have shown that the diversity of PGTCPs extends far beyond leopards and a few other animals, to thousands of species, including angiosperms. In addition, while some of the effects of growth were already known (like rosette formation), we used simulations to show other possible effects that had not yet been described and were biologically plausible. We then produced a large number of concordant clues of different kinds, showing the very high probability of growth as the cause of PGTCPs. As the great diversity of PGCTPs may suggest potential biological functions, we have described and discussed several of them, with supporting arguments”*

—---------------------------------------------------

***Reviewer #2: The concept of reaction-diffusion mechanisms as a means to explain periodic patterns in nature has been prevalent for over seven decades. This elegant mathematical theory proposed by Alan Turing stands as a cornerstone in our understanding of natural patterns. The authors of this study have honed in on a particularly pervasive yet specific phenomenon: the coupling of reaction-diffusion patterning with tissue growth, termed here as 'putative growth Turing-like color patterns' (PGTCPs).***

***This paper is commendable for its unique perspective on how tissue expansion influences reaction-diffusion patterning. It delves into several intriguing facets of this subject. The authors commence their exploration with a comprehensive, large-scale screening to determine the prevalence of PGTCPs across various species. This is followed by an insightful elucidation of the formation mechanisms underlying four distinct sub-classes of PGTCPs. The comparative analysis of PGTCP manifestations in different parts of the same organism, in relation to the varied tissue growth dynamics at these sites, is particularly noteworthy. The hypothesis presented towards the end, suggesting the evolutionary role of PGTCPs in signaling and camouflage, adds an intriguing dimension to the study.***

***However, as a manuscript intended for a general readership, it requires further refinement. The figures and explanatory sections particularly need enhancement for better clarity. Providing clear, concise key information and essential details is crucial to aid readers in grasping the narrative flow and the methodologies employed in the experiments. Such improvements would significantly augment the paper's accessibility and comprehension.***

***Major revisions:***

***A fundamental question addressed in this study is the dependency of the final pattern outcome on the rate of tissue growth relative to the dynamics of pattern formation mediated by the Turing mechanism. The authors propose a hypothesis regarding the formation of four typical putative growth Turing-like color patterns (PGTCPs), suggesting that tissue expansion plays a decisive role only during a specific phase of patterning. According to this hypothesis, there are three distinct phases: Phase I: The tissue remains unchanged, allowing the initial pattern to form and stabilize.***

***Phase II: Tissue expansion becomes significant, catalyzing further evolution of the pattern and the emergence of PGTCPs. Phase III: Tissue expansion slows down or ceases entirely, preserving the newly emerged PGTCPs without further alteration. This critical explanation is currently located in the legend of Supplementary Figure 1C. However, given its importance in understanding the interplay between tissue growth and patterning, it would be more appropriate to incorporate this discussion into the main text, preferably in the early sections of the manuscript.***

Thanks for this important remark.

As the literature is very exhaustive concerning the mathematical approach on Turing pattern formation, I decided not to focus to much on dynamics, also for two other complementary reasons: 1) we usually simulate Turing patterning with reaction-diffusion equations with two morphogens, but the real biological systems could be very different and much more complex, leading to any discussion on dynamics partly obsolete. Because I wanted to focus on biological clues in this article, I generally tried to avoid discussion about mathematical relationships. 2) The other reason is that growth could generate PGTCPs under many conditions. For example, in the study, I decided in simulations to begin with a first phase of Turing pattern formation without tissue growth, then start growth and observe the effects. Nevertheless, this first phase of tissue size stasis is not mandatory at all. Under particular conditions (notably if growth is not too fast compared to Turing dynamics), we could implement growth directly at the beginning, and PGCTPs could also arise. Also, concerning the growth rate dynamics, many different conditions (e.g. linear or exponential growth) can lead to the same final pattern. Then, I did not want to discuss all these possible conditions too much, but just to show that growth is sufficient to generate the patterns.

To explain this choice, and to clarify things, I added explanations in the part 2, together with adding details in Figure 2 (see below in Minor revisions).

-> lines 270 to 273 *“In all of our simulations, in order to simplify, we chose to let the Turing-like system to start in a non-growing domain, but it is not a mandatory condition. Then, at one point, growth starts, and we chose to make growth rate constant so the tissue surface grows exponentially, which is classical during development.”*

***In the 'Materials and Methods' section, particularly in the PGTCPs modeling session, it is imperative for the authors to provide essential information about the models. This should include the modeling assumptions, master equations for each circuit species, simulation methods, and expected outputs. Such details are crucial for comprehensively understanding and replicating the study's findings.***

Thanks for this useful remark. Together with remarks of Reviewer 3, I decided to build a new model incorporating the effects of growth in diluting the concentrations of the agents. Therefore, I took this opportunity to describe more precisely the model, in the text in Materials and Methods. This information was previously available in the code, but now it is also available in the article text.

->lines 608 to 625 ”*Master equations:*

*a(t+1)=(a(t))/(1+g*dt)+(16-(a(t))/(1+g*dt)*(b(t))/(1+g*dt) + (d(t))/((1+g*dt)*(1+g*dt) )*r*s*∇2a),; dt*

*b(t+1)=(b(t))/(1+g*dt)+((a(t))/(1+g*dt)*(b(t))/(1+g*dt) - (b(t))/(1+g*dt) – 12 + (d(t))/((1+g*dt)*(1+g*dt) )*s*∇2b)*dt*

*Where a and b are the morphogens concentrations, g is the growth rate (of the surface), r and s are ratio and scale parameters of De Gomensoro Malheiros et al equations (20), ∇2a and ∇2b the Laplacian operators. The discrete Laplacian operator used in the simulations is the standard nine-point stencil, covering a Moore neighborhood of unitary size: [ [1, 4, 1], [4, −20, 4], [1, 4, 1] ]/6.*

*The code of master equations:*

*float newvaluea = oldvaluea/(1+g*dt) + (16 - oldvaluea*oldvalueb/((1+g*dt)*(1+g*dt)) + d*r*s*laplaceA)*dt;*

*float newvalueb = oldvalueb/(1+g*dt) + (oldvaluea*oldvalueb/((1+g*dt)*(1+g*dt)) - oldvalueb/(1+g*dt) - 12 + d*s*laplaceB)*dt;*

*where g is the growth rate. Growth was indirectly implemented by its effect on diffusion (via s parameter) and its effect on morphogens concentration (via dilution, on parameters a and b).*

*g is equal to zero until a certain point (t = 10000 in all of our simulations), meaning that a classic Turing system starts to organize before growth starts.”*

Besides, we add a table with all variables for each simulation presented in Figure. 2.

-> lines 633 to 634 “*Variable parameters for simulations presented in this study:*

| *Simulation* | *Leopard* | *Emu* | *Pomacanthus* | *Ictidomys* | *Tetraodon* |
| --- | --- | --- | --- | --- | --- |
| *Initial conditions* | *Random noise* | *One middle stripe* | *One middle stripe* | *Periodic stripes* | *Random noise* |
| *s* | *6* | *10* | *10* | *6* | *10* |
| *r* | *30* | *6* | *6* | *30* | *8* |
| *g* | *0.05* | *0.05* | *0.05* | *0.01* | *0.075* |
| *dt* | *0.002* | *0.002* | *0.002* | *0.002* | *0.002* |
| *Timing of*  *growth start* | *10000* | *10000* | *20000* | *1000* | *10000* |

*“*

***Minor revisions:***

***In the figure legends, the authors should provide more comprehensive details about the key steps of each simulation experiment to facilitate a better understanding of the simulation results among readers.***

Done. In every simulation presented in the Figure 2, I had details in every screenshot and in the Figure captions, in a complementary manner to the Material and Methods text.

***Figure 1A:***

***Do these four categories encompass the entire spectrum of PGTCPs?***

Thanks for this comment. They encompass all the spectrum of new geometries we described for PGTCPs.

For more clarity, I precise that these categories are exhaustive (in my current knowledge) in the Figure captions and in the plain text

-> lines 126 to 127: “*The PGTCPs were classified in an exhaustive list of four categories,”*

***Figure 1C & 1D:***

***What criteria are used to differentiate classic Turing patterns from PGTCPs? Is this classification based on visual assessment of stable patterns in standard adult species? For instance, are spots, lines, and mazes classified as classic Turing patterns, while rosettes, line-and-dot alternations, mixed colors, and intermediate bands are categorized as PGTCPs?***

Thanks for these questions. PGTCPs are all putative Turing-like patterns (*i.e.* periodic and not relying on pre-existing periodicity) which can not be reproduced by classic Turing simulations, whatever the system parameters are. So exactly: spots, lines and mazes are then classic Turing patterns, while rosettes, line-and-dot alternations, mixed colors, and intermediate bands are categorized as PGTCPs.

Unfortunately, we cannot use adult stable patterns to define classic Turing patterns, because in most species the Turing system is stopped at one time during the development and the pattern is screenshot. For this reason, many adult “stable” patterns are PGTCPs “frozen in time”.

Together with the first remark of Reviewer #1, this brought me to add a more complete and precise definition of PGTCPs in the beginning of the manuscript, in the introduction section:

-> lines 87 to 91 “*Here, we consider a particular color pattern as a PGTCP when it possess all of these characteristics: i) periodic, at least at the local scale, ii) non-based on a pre-existing periodicity (e.g. body segments or petals), iii) with at least three motifs (e.g. three stripes or rosettes) and iv) with at least one motif not belonging to the classical range of Turing geometries (dots, stripes, mazes), i.e. line-and-band alternations, rosettes for example.”*

***Could you clarify the distinction between the categories “Yes” and “Maybe”?***

Done. This was described in Supp. Figure 3.C but thanks to this suggestion I clarified it much more earlier in the manuscript and in Figure 1 captions.

-> lines 152 to 153 “*The criteria for the classification into each category (PGTCPs, Maybe PGTCPs, classic Turing, Maybe classic Turing, none) are detailed in Supp. Fig. 3*.”

***Figure 2A:***

***Can you describe how the tissue expands over time? Does it begin expanding constitutively from t = 0?***

***Intuitively, a very slow expansion rate might lead to a regular spot pattern. If the expansion rate is significantly higher, how does the rosette pattern evolve dynamically? Do "disrupted rosettes" emerge as a pattern?***

Thanks for this remark.

Concerning the first part of the question, I added much more precision in the Figure 2, because it was only available in the simulations codes. Furthermore, I precised every starting timings in the parameters table added in Material and Methods (see above). I tried different starting timings for growth, and it does not affect the results of simulations much (as long as growth rate is not too high), so I have chosen not to discuss this particular point in the main text of the article.

For the second part of the question a very slow expansion rate does not (necessarily) mean the absence of PGTCPs. After the generation of a first generation pattern, when the tissue will reach a larger size, motifs will typically start to disrupt, for example dots will become rosettes) and during the process, PGTCPs can be observed.

On the contrary, a too high expansion rate will generate a strong dilution of morphogens and prevent the random noise from reaching sufficiently high values to transform into motifs. Alternatively, if strong growth starts after a primary classic Turing pattern, it might erase it by diluting.

***Figure 2C:***

***What is the time sequence for the six panels shown?***

***How does tissue expansion correlate with pattern formation dynamics?***

Thanks for these questions. Time sequence (and the start of growth) are now precised for all simulations with a red arrow in Figure 2, with the screenshots from simulations done with the new model.

To have a more precise view of the relations between tissue size dynamics and pattern formation dynamics, and in particular the delay of PGTCPs apparition, please see Supp. Figure 1C: in dashed lines is the expected number of motifs, which is proportional to tissue size, and in solid lines are the real number of patterns observed.

***Figure 2D:***

***What does the dotted line plot represent? Below the lower t = 0 panel, two color sets are used for the dots. Please specify their meanings.***

Thanks for these questions. They represent the values of the activator morphogen concentration in the middle horizontal transect, in a complementary way to the color code. They were removed in the last version of the Figure, because they were not sufficiently informative and difficult to read. Nevertheless, they can still be produced during the simulations running, with the code available online.

***The dotted line plot below the second row is too dim to discern.***

***In the lower t = 0 panel, are the stripes pre-assigned as initial conditions for the simulation?***

I agree, they were then removed from the Figure, but still available when simulations are run with the code available online.

In this example, stripes are pre-assigned as a simplification, but it is a non-mandatory step, as shown in several articles, notably De Gomensoro Malheiros et al. 2020 (<https://dl.acm.org/doi/abs/10.1145/3386569.3392478>), or in Figure 2.C (emu modeling).

***Is it correct to assume that in these simulations, tissue expands only along the x-axis?***

Thanks for this question. The directionality of stripes prevents “observing” the y-axis elongation, but it is still present. In every simulation, except for Figure 1.A (*Phalaenopsis hieroglyphica*), growth was chosen as homogeneous in every direction (although the real calculations used an approximation with the Laplacian operator), for simplification.

***Figure 2F:***

***This figure appears to have the same issues as mentioned for Figure 2C.***

Thanks for this remark. Time sequence (and the start of growth) are now precised in Figure 2, with the screenshots from simulations done with the new model.

***Figure 3B:***

***Please specify what the star marks and lines indicate.***

Done (in figure 3B caption).

*lines 312 to 313 “Stars are the homologous landmarks, used in geometric morphometric studies, lines are the homologous outlines of the organism bodies.”*

***Supplementary Figure 1C:***

***Label the y-axis to provide clarity***

Done, thanks for this remark.

—---------------------------------------------------

***Reviewer #3, Dr. Andrew Krause:***

***The author explores the role of domain growth in modulating Turing patterns, leading to ‘non-classical’ structures like stripes and dots, as well as rosettes. They survey a wide range of taxa from different phylogenies of Eukaryotes, finding that some lineages have variation in the occurrence of ‘classical’ Turing patterns (spots, stripes, mazes), as opposed to ‘putative-growth Turing color patterns’ (PGTCPs). Beyond these classifications, they build a simple model of a growing reaction-diffusion system, and perform a range of analyses between the observed morphologies and the simulated patterns. They include a nice discussion of plausible mechanisms regarding pattern fixation or disturbance, providing some interesting hypotheses regarding the role of growth (and other factors, such as memory and cell-fate fixation) in originating a diversity of patterns. They also consider a mechanism-based way of understanding the origin of these patterns given morphometric data.***

***Overall I find the topic interesting, and the core ideas of surveying wide taxa and matching observations with plausible theory very interesting. However, I have a number of serious concerns about the theoretical aspects of the work, largely to do with how the mathematical models are set up and how they are interpreted. I think the ideas in this paper are interesting, but from my point of view much more work is needed to make a significant contribution to the literature. Much of the analysis is broad but shallow, with some important caveats in reference to prior work on growth, pattern robustness, etc. Below I outline a number of suggestions for improvement. It should be noted that I do not think the authors must address every comment below in copious detail – rather, I feel they should consider telling a deeper story with more significant insights, focusing either less on specific mechanisms (and hence more qualitatively on the observations), or more narrowly justifying a specific mechanism such as growth in the formation of complex patterns.***

Thanks for these comments and remarks. I hope all the changes mentioned above and below will address your main points.

**Key Comments**

**1. Growth is an Inadequate Mechanism for Pattern Modulation: As far as I understand the current manuscript, the author ascribes patterns which do not fall into a classification of spots or labyrinthine structures (stripes or mazes) as putatively due to growth modulating a Turing (reaction-diffusion) mechanism on the level of gene expression (or plausibly cell phenotypes or another mesoscopic structure). However, growth itself requires a fairly precise mechanism of fixation to transform a reaction-diffusion pattern into a fixed morphological structure. This fixation is nontrivial. In fact, even just considering transient pattern formation alone (i.e. allowing a modeller to ‘fix’ a transient pattern at any stage), one can essentially generate any possible observed pattern given freedom over the nonlinearities and initial conditions.**

**Perhaps more importantly, there are many other mechanisms which can also generate all of the ‘non classical’ patterns described by the author. In particular, work combining ideas of positional information and Turing patterning (Green and Sharpe, 2015; Krause et al., 2020) emphasises that spatial modulation and simple ‘pre-patterns’ can generate structures such as rosettes and stripe-to-spot transitions within the domain. See Woolley et al. (2021) for a demonstration of how spatial and/or temporal forcing can give rise to essentially any pattern or sequence of observed patterns in reaction-diffusion systems. Even in spatially homogeneous (unforced) systems, one can find a huge range of different pattern behaviours, depending**

**on the system’s nonlinearities (presumptive reaction kinetics) and initial conditions; see (Pearson, 1993; Kondo and Miura, 2010) for several examples.**

**Overall, I am not at all convinced that structures such as rosettes must be due to growth, as they cannot be from growth alone but must involve a carefully-time fixation mechanism for them to survive beyond the embryonic stage. Additionally, I think there are several other plausible hypotheses regarding pattern modulation that more parsimoniously provide plausible mechanisms for such ‘non-classical’ patterns. I like the database that the author has built, and I do think such classification is valuable, but I would be substantially more hesitant in claiming that growth is the key modulator of patterning.**

Thanks very much for these very detailed comments. Please find below my answers for every point mentioned in this part:

First, I do agree qualitatively with Dr. Krause that growth might not be the only mechanism generating the motifs called PGTCPs in this manuscript. Alternative mechanisms are possible, are well described in the literature and I thank Dr. Krause for bringing to my attention some of them that I didn't know. Therefore, I mentioned them in the introduction part and added a reference:

-> lines 75 to 77 “*Most of those models perfectly reproduced leopard rosettes by adding the most probable missing ingredient: tissue growth during pattern formation. Other types of missing ingredients have been described, like complex pre-patterns (21).”*

However, I think our opinions diverge quantitatively concerning the role of growth. While Dr. Krause sees growth as “an Inadequate Mechanism for Pattern Modulation”, and might not be responsible for the majority of non-classic Turing-like patterns, after this study I am now convinced that growth might account for more than 99% of the PGTCPs. This is the subject of Figure 3, which summarizes several independent arguments, and I would like to precise some of them here.

**“However, growth itself requires a fairly precise mechanism of fixation to transform a reaction-diffusion pattern into a fixed morphological structure. This fixation is nontrivial.”**

I do agree with the first sentence, but I might be more prudent with the second one. In cell biology, fixation of cell and tissue phenotypes are very common and well described, and it relies on many mechanisms, like epigenetics. Concerning color patterning, when colored cells like melanocytes begin to transition to their final coloration (producing eu- or pheo-melanin), it is non reversible. However, pattern modulation might arise but with other cells not yet differentiated.

The main reason why I do not see pattern fixation as a major issue is the tens of thousands examples of species exhibiting distorsions on their periodic color patterns. For example, zebra juveniles are born with their final pattern, no stripe will be added or removed, but during its growth, as their body parts will know allometric growth, some stripes will be distorted compared to others (typically on the back compared to the head). This is observable in many species, including many animals and almost all plants. In plants, Turing systems seem to stop quite early during development so the resulting patterns undergo distorsions during petal (or other structures) allometric growth. It is particularly the case In the only group where a Turing-like system has been explored at the molecular scale (*Mimulus* genus).

Distorted patterns are then a strong clue that pattern fixation might occur very easily in many different species.

Nevertheless, it is not sufficient to suggest that growth might account for >99% of PGTCPs. To affirm this, I produced several types of independent arguments and simulations, which were the subject of the third part of the manuscript (Figure 3). The strongest clue might be the absolute correlation between the most growing zones of the organisms and the presence of PGTCPs. For a few species, I produced a morphometric tool to compare growing zones and PGTCPs occurrence (Figure 1B), then I showed that for all the Feliformia species (the most important group of mammals exhibiting PGTCPs) there is a perfect correlation between PGTPCs and high growth zones, and finally, after the observation of around more than ten thousands species of animals, and more than 40000 species of plants, I have never observed a single example of a presence of PGTCPs in (what it is traditionally considered as) a low-growing zone **together** with the presence of only classic Turing-like patterns in a high-growing zone. Therefore, after all these observations in many independent taxa, I am compelled to consider growth as the most parsimonious candidate to explain the presence of PGTCPs.

The only caveat to these observations is that correlation is not direct causation, and that maybe growth is responsible for PGTCPs but in an indirect way, different to the tissue scale effect. In this hypothesis growth (1) causes an unknown effect (2) and this effect generates Turing pattern modulation (3). Anyway, in the current state of knowledge, it might not be a trivial hypothesis, as it implies the fact that we have to explain i) what could be this alternative mechanism, ii) why Turing patterns are not sensitive to tissue scale change by growth, and iii) how it could be present in a very large diversity of taxa. Alternatively, we might think of a mechanism causing both growth and PGTCPs formation, leading to a correlation, but again, it might not be considered as a trivial hypothesis.

**2. Implementation of Domain Growth: The model presented in (20) and implemented by the author in Processing neglects an important physical effect of growth. Namely, changing only the diffusion coefficient does not produce equations which satisfy conservation of mass as the domain size changes. To see this, note that changing the diffusion coefficient does not change uniform concentrations/spatially homogeneous equilibria. Assume such an equilibrium is in a domain which is growing. Then additional mass must be produced in order to maintain the same concentration everywhere on a larger region of space. This was an error in the original edition of the book (Murray, 2004), though was corrected by Crampin et al. (1999, 2002) and many others since. See, for example, the models in Seirin Lee et al. (2011); Van Gorder et al. (2021); Krause et al. (2023) which all include an extra term referred to as ‘dilution.’ For sufficiently slow growth, this term will be small, but it will still have an impact on the transition between patterns, the stability of patterns, etc.**

**In the case of a domain given by x ∈ [0, L(t)] which is growing uniformly in time, then the equations the author should instead simulate look like:**

**_∂t =_^1^L^2^ D∇^2^**

**∂u**

**Diffusion**

**u + f(u)**

**Reactions**

**_−L_^˙^L**

**Dilution**

**_, L_^˙^ _=_^dL^_dt_ ,**

**where u is a vector of concentrations, D is the matrix of diffusion coefficients, f are the reaction kinetics, and the final term arises due precisely to this domain growth effect. A very rudimentary version of the author’s model with domain growth can be found at the URL in this footnote^1^ using the Visual PDE website for simulating interactive PDE-based models. I don’t think this exactly captures the physics, but one can see the growth-rate dependent behaviour due to this dilution term by increasing the growth rate parameter r – above some threshold, patterning is suppressed. Perhaps more importantly, the author can explore many of the simulations on this website that exhibit a variety of Turing-like patterns and related mechanisms of pattern formation.**

Thank you for this very important remark. Thanks also for bringing my attention to the very useful website which allows to easily simulate PDE.

In the previous simulations, the effect of growth in morphogens dilution was ignored, or considered as negligible, but I agree that it is not the case for high growth rates, which could in particular make the emergence of new motifs from noise difficult. To do more realistic simulations, I modified my model by adding an effect of growth on morphogens concentrations, and redid all the simulations of Figure 2. As similar results were obtained (due to the low growth rate used in these simulations), others Figures containing simulations (Figure 3, 5, Supp. Fig. 1 and Supp. Fig. 2) have been kept unchanged.

New model changes have been detailed in both plain text, Material and methods, and of course in the code (see below, response to Reviewer #2 for details).

**3. Connection to the Theoretical Literature: As suggested above, I think the author has done an excellent job surveying a range of biological literature, but has missed crucial insights discussed in more theoretical papers. In particular, the original work of Crampin et al. (1999) was to ameliorate robustness issues in simple models of pattern formation; see Maini et al. (2012) for discussion of these issues, and the only partial progress made by introducing growth. As noted above, a major disadvantage of reaction-diffusion models on growing domains is how to ‘fix’ the pattern in time, providing another degree-of-freedom which needs tuned and invoking additional underlying mechanisms. Robustness is one of the key challenges even for simple/classical patterns; see Scholes et al. (2019) for further discussion of this point. I think one has to tread carefully in describing robust complex behaviour, as sufficiently complicated models can exhibit almost anything we’d like to see.**

Thanks for this complementary remark (and the additional references) on the subject of fixing growth effects in patterns. Please see above for the point of view (biodiversity-based) chosen in the manuscript.

**Other Comments**

**4. The author states that Turing patterns have been observed in vivo and are responsible for many structures observed in biology. I would argue that almost all cases like this as presumptive; typically far more than two genes are implicated in these studies, and it is not perfectly clear that the mechanism is one of activator-inhibitor type. While the mechanism is plausible, I would be more cautious about its ubiquity across Eukaryotes. Even in work I was involved in (reference (13)), I think we are somewhat cautious to note that the reaction-diffusion mechanism is plausible, but that there are distinct modulations of the simple theory not entirely captured by simplistic two-species models. As an example, there has been recent work unraveling the more complex pattern formation in Zebrafish that goes beyond simplistic activator-inhibitor thinking (Bullara and De Decker, 2015). Even Shigeru Kondo, who authored reference (10), has since suggested important caveats to any simple reaction-diffusion model of Zebrafish (Kondo et al., 2021).**

I do agree that real biological systems might be much more complex that the simplistic two-morphogens (activator+inhibitor) model. Of all the systems currently explored, the *Mimulus* system might be the closest to the classic minimal system, as it is based on at least one diffusive molecule, and two main genes seem involved, with activation and inhibition relationships compatible with the classic Turing-like system. But it is clear that many more agents might add their effects to the system. What is also clear is that when studies try to decipher the cellular/molecular bases of “Turing” periodic patterns, all the systems seem (but only seem) compatible to “Turing-like” systems. It is an argument that needs to be taken with caution, because it is possible that there are biases (typically, you might only find what you look for). Therefore, I added precaution during the presentation of Turing systems in the Introduction (see remark 5).

**5. Line 66 is missing a paren around “(spots stripes and not much more).” I would also suggest that reaction-diffusion systems can exhibit an enormous diversity of patterns and structures when one moves away from the classical minimal model systems (Pearson, 1993; Woolley et al., 2021).**

Thanks for the remark and the references. The context of this assertion “spots stripes and not much more” was classic Turing patterns, but I make it more clear for the reader, together with the precedent remark.

-> lines 63 to 67 :” *After years of mathematical formalization (8) and modelling (9), compatible patterning systems were described in vivo (10) and might be responsible for many biological repeated structures called Turing-like patterns. Characterized by their periodicity and the range of repeated motifs shapes (spots, stripes, mazes and not much more in the classic Turing-like systems (11)),”*

**Minor Comments/Typos**

**6. Many of the references are incorrect and inconsistently typeset. For example, (29) is missing an author and is written in capital letters unlike nearby references.**

Done. Thanks for the remark. All references were corrected and highlighted in yellow in the manuscript (36, 31, 39, 44, 52)

**7. The URL “eukolorpatterns.cnrs.fr” is misspelled with an @ symbol rather than a . before “fr.”**

Corrected. Thanks.

**NB: The manuscript already cites many references, so please do not feel the need to include any of the references I have added (especially any I am an author on). Rather, I am merely hoping the author considers some of the perspectives presented in these papers which may have been overlooked.**

Thanks for these references, some of them are very complementary to the already mentioned papers, so I took the opportunity to cite them in the manuscript.

—-----------------------------

End of rebuttal letter

Pierre Galipot

March, 28th 2024
