# Supplementary material for: And growth on form? How tissue expansion generates novel shapes, colours and enhance biological functions of Turing colour patterns of Eukaryotes
Source: PLoS One. 2025 Feb 3;20(2):e0305921. doi: 10.1371/journal.pone.0305921 (PMC11790173; doi:10.1371/journal.pone.0305921)
Supplement: Attachment — Submitted filename: Response to Reviewers.docx [file pone.0305921.s006.docx]

To : the Editors of PLOS One	At Rennes, May, 31st 2024

Subject : Rebutal letter

Dear Editors,

I would like to thank the reviewer for his/her comments on the manuscript and I have edited the manuscript to address him/her concerns. I believe and hope that the manuscript is now suitable for publication in PLOS One.

Please find below my answers to every point mentioned by the reviewer. His/her reviews are in bold and the answers in yellow-brown. Quotes of corrected texts are in yellow-brown and in italics.

I remain at your disposal for any information,

Very sincerely,

Dr. Pierre Galipot

ECOBIO

Université de Rennes - Campus Beaulieu

Rennes, FRANCE

***Journal Requirements:***

***Please review your reference list to ensure that it is complete and correct. If you have cited papers that have been retracted, please include the rationale for doing so in the manuscript text, or remove these references and replace them with relevant current references. Any changes to the reference list should be mentioned in the rebuttal letter that accompanies your revised manuscript. If you need to cite a retracted article, indicate the article’s retracted status in the References list and also include a citation and full reference for the retraction notice.***

Thanks. The reference list has been reviewed, and no issues concerning potential retracted articles have been found.

***Additional Editor Comments:***

***Dr. Woolley raised an important point that the definition of PGTCP is not clear. In my opinion，the important message in this manuscript is that growth can modify or accelerate Turing patterning formation to create diverse morphologies in the Eukaryotes. In a simple word, growth is an important variable in the Turing patterning formation over time. I think the definition of PGTCP should be simplified and revised. It might be better to refer it simply as "growth accelerated Turing patterning".***

Thanks for these useful comments. Please find below, in the response to Reviewer #1, the changes I have made to make the definition of PGTCPs clearer. I have considered many names for these particular patterns during the writing of the paper, and no one was completely satisfying both in terms of shortness and precision. After taking advice from various researchers both in morphogenesis and outside, I chose the acronym PGTCPs, which has its limits in terms of simplicity, but it encompasses all the necessary (and sufficient) characteristics of these patterns: i) the fact that we make the hypothesis that growth is responsible for them but that functional proof lacks “PG”, ii) the fact that we make the hypothesis that they are modified Turing-like patterns “P…T…Ps” and iii) the fact that we chose to restrained the study to color patterns, “C”, because Turing-like systems are able to produce non-color patterns. When functional studies would -hopefully- bring the proof that growth is really responsible for PGTCPs, or at least some of them, I totally agree that we might use a simpler name. I apologize for not being able to respond to your suggestion to find a simpler name that would still be informative.

***Reviewer #1: The author has addressed my comments, but I cannot say I'm satisfied by their respeonse.***

***My first criticism was a lack of definition. They have no added one, but if anything it strengthens my criticism. The definition is***

***"Here, we consider a particular color pattern as a PGTCP when it***

***possess all of these characteristics: i) periodic, at least at the local scale, ii) non-based***

***on a pre-existing periodicity (e.g. body segments or petals), iii) with at least three motifs***

***(e.g. three stripes or rosettes) and iv) with at least one motif not belonging to the***

***classical range of Turing geometries (dots, stripes, mazes), i.e. line-and-band***

***alternations, rosettes for example."***

***Firstly, this moves the problem onto defining what a motif is. Moreover, the first two are base properties of Turing patterns,***

Thanks for your comment. The first two are indeed properties of Turing patterns, and it is not a coincidence, as this paper hypothesis is that PGTCPs are Turing-like patterns modified by growth (the “T” of PGTCP).

***(iii) is completely arbitrary (why three motifs?)***

I agree, this is partly arbitrary, but we chose to impose a minimum number of motifs for the following reason: when the number of motifs is low, there is a high probability that other types of mechanisms than Turing-like could produce them. For example, there is “no need” for a Turing system to produce a single stripe, we can find many other mechanisms in the litterature. We then decided to choose 3 motifs as a compromise, but we do not exclude the possibility that PGTCPs with only one or two motifs exist in nature, and alternatively that some of the three- or four-motifs patterns we have found might not be produced by a Turing-like system (This is why we chose the first “P” in PGTCPs, for Putative). For the same reason, we chose to exclude repeated patterns based on a pre-existed repeated geometry. For example, if every petal of a flower harbors a single dot, then of course the flower presents kind of a repeated color pattern, but in terms of probability that a Turing system produced it, it is the same “one-motif” case evoked earlier.

All the criteria were detailed in the Supp. Fig. 3 but we added some precision in the Figure legend and in the Material and Methods section.

lines 569 to 577: *“We chose to impose a minimum number of motifs to consider it as a PGTCP for the following reason: when the number of motifs is low, there is a high probability that other types of mechanisms than Turing-like could produce them. We decided to choose 3 motifs as a compromise, but we do not exclude the possibility that PGTCPs with only one or two motifs exist in nature, and alternatively that some of the three- or four-motifs patterns we found might not be produced by a Turing-like system. For the same reason, we chose to exclude repeated patterns based on a pre-existed repeated geometry. For example, if every petal of a flower harbors a single dot and therefore an overall periodic color pattern, a Turing mechanism might not be needed to produce it.”*

lines 1007 to 1009, in the Supp. Fig. 3 caption: *“A minimal number of three motifs is required to consider it as a good candidate for a PGTCP, as one or two motifs patterns might be produced by other mechanisms than the Turing system.”*

***and (iv) simply says it doesn't look like a Turing pattern. Nowhere in the definition is growth required, thus, many of the rebuttal points which are of the form: they don't include growth, so they're not PGTCPs, are invalid.***

Thanks for this comment. Growth is not required in the definition because this analysis is based on pictures observation, so no data on growth rates are available (This is why we chose the first “P” in PGTCPs, for Putative). Of course, if we have access to the information that the tissue experiences no growth during pattern formation, we could exclude that this is a PGTCP. To my knowledge, this information is not yet available for any species bearing PGTCP, but to be honest, this is the subject of the next project I am working on.

***In particular, most of the papers I've suggested in my previous review fit this description. It simply says that they're patterns that don't fit the normal Turing stereotypes, of which there is a lot of literature, which is currently being ignored.***

***Beyond that the conclusion is no stronger. The conclusion is still: Turing patterns can account for some patterns but not all. To achieve more complexity we need to add more complex mechanisms. This is known and the paper doesn't really add any conclusion beyond growth can change the produced Turing patterns.***

Thanks for this comment. To begin with, I agree that there is a lot of literature on non-classic Turing patterns, and much of it showed that growth is able to disrupt Turing systems and to generate non-canonical motifs. This is not the main subject of the article so it is normal that it doesn’t change the conclusions of the literature much. Nevertheless, in the modeling part summarized in Figure 2, we showed an alternative way on how growth can induce non-canonical motifs, which wasn’t -as far as I know- already described in the literature. Moreover, we explained how this or that effect of growth is adapted to this or that biological system (*e.g.* memory mechanism is adapted to the leopard, Fig. 2B, and the new mechanism is more adapted to the *Pomacanthus* Fig. 2D). In summary, growth could have effects but through several mechanisms.

If our article was restricted to this modeling part, I would agree that “***the paper doesn't really add any conclusion beyond growth can change the produced Turing patterns***”.

However, the article presents many other aspects of Turing patterns and growth which go beyond this conclusion. Please let me rephrase the main conclusions of each part in a very summarized way:

Part 1/Figure 1: PGTCPs (i.e. patterns that might be produced by the interaction between growth and Turing systems) are present all over the Angiosperm and Animalia phylogenetic tree. From 4 or 5 species already described in animals, and no species mentioned on flowering plants, this article brings thousands of new species, representative of all the diversity. This large diversity could suggest specific biological functions for PGTCPs.

Part 2/Figure 2: Modeling shows that growth could affect Turing patterns in many ways (including a novel one), and can account for all non-canonical Turing patterns in nature.

Part 3/Figure 3: PGTCPs occurrences analysis and morphometrics show that PGTCPs coincide with most-growing regions of the bodies, strongly suggesting that growth is responsible for the majority of them.

Part 4/Figure 4: Additionally to the biological functions of classic Turing color patterns, PGTCPs could allow individuals to be less visible (notably through mixing colors) and more visible (notably through the production of fractal motifs and with motifs not in the abiotic environment).

—-----------------------------

End of rebuttal letter

Pierre Galipot

May, 31st 2024
